# Clinician Specialties, Quality Score and Shared Savings Receipt in Accountable Care Organizations

**DOI:** 10.1111/1475-6773.70033

**Published:** 2025-09-04

**Authors:** Mariétou H. Ouayogodé, Xiaodan Liang

**Affiliations:** ^1^ Department of Population Health Sciences University of Wisconsin School of Medicine and Public Health Madison Wisconsin USA

**Keywords:** ACO, medicare, provider composition, quality of care, shared savings program

## Abstract

**Objective:**

To assess the relationship between the changing Accountable Care Organizations‐ACO workforce and ACOs' shared savings earnings and quality performance.

**Data Sources:**

Medicare Shared Savings Program‐MSSP provider‐level research identifiable files, performance year financial and quality report public use files, and National Physician Compare data (2013–2021).

**Study Setting and Design:**

We characterized 865 MSSPs, separately pre‐ (2013–2019) and post‐pandemic (2020–2021) according to the percentage of primary care physicians (PCPs), non‐physicians, specialists, and other specialty, financial risk model, assigned Medicare beneficiary demographics, clinical risk factors, and provider supply by specialty within the MSSP's primary service state, (total and per‐capita) shared savings earnings/losses owed and quality score. Longitudinal ordinary least‐squares regressions with random effects were estimated to assess the association between MSSP provider specialty mix and annual (1) per‐capita shared savings/losses and (2) quality score, controlling for risk model, beneficiary characteristics, provider supply, and year factors. We also compared outcomes across MSSPs, 32 Pioneers and 62 Next Generation‐NGACOs.

**Principal Findings:**

PCPs represented 33.9% of MSSP's workforce, on average. Higher percentages of PCPs and non‐physicians were associated with higher per‐capita earned shared savings and quality scores among MSSPs. A 1‐percentage‐point (ppt) increase in PCPs and non‐physicians was associated with higher per‐capita shared savings of $2.25 (*p* < 0.01) and $1.82 (*p* = 0.03), respectively, pre‐COVID, and $2.73 (*p* < 0.01) and $1.81 (*p* = 0.14) post‐COVID. We estimated increases in quality scores among MSSPs of ~0.1 ppt with a 1 ppt increase in PCPs, non‐physicians, and specialists only pre‐pandemic. No statistically significant relationships were estimated between provider specialty mix and performance measures in Pioneers and NGACOs.

**Conclusions:**

Higher percentages of PCPs and non‐physicians were associated with higher per‐capita shared savings earnings and quality scores among MSSPs. As new federal initiatives continue to unfold, value‐based payment models increasing incentives for primary care should be monitored to determine their ability to further improve care efficiency.

**Summary**

- What's known about the topic
  ○ The healthcare provider landscape has been changing among ACOs with a decreasing percentage of primary care physicians and a growing percentage of non‐physicians participating in these payment models.
  ○ The change in provider compositions may result from the eligibility of non‐physicians (nurse practitioners and physician assistants) to enter the first stage of the ACO patient attribution process in 2016.
- What this study adds
  ○ This study assessed the association between ACO providers' specialty distribution, ACOs' quality of care, and shared savings/loss payments during the first decade of Medicare ACO programs.
  ○ Primary care physicians and non‐physicians represented nearly 60% of the provider workforce in the largest and permanent Medicare Shared Savings Program (MSSP).
  ○ ACOs with higher percentages of primary care physicians and non‐physicians achieved higher per‐capita earned shared savings and quality scores only in the MSSP.

## 1 Introduction

The healthcare workforce among participants of the Medicare accountable care organization (ACO) model has changed over the years, with a decreasing proportion of primary care physicians [1]. Medicare ACOs emphasize primary care and coordination of care across the continuum. Since 2012, healthcare organizations have been entering Medicare ACO contracts that hold them accountable for total cost management and some quality metrics for their patients. Participants with attributed patient spending below their financial benchmarks can earn a share of these savings back, conditional on meeting standards in the quality of care. In Medicare ACO contracts, patients are attributed to participating organizations based on their use of primary care services from eligible providers. The eligibility of non‐physicians (nurse practitioners and physician assistants) into the first stage of the patient attribution process in 2016 has opened more opportunities for these clinicians to impact care delivery in ACOs [2].

Prior research in the first performance year of the Medicare shared savings program (MSSP) participants determined that the organization's leadership structure and prior experience with risk‐bearing contracts may be positively correlated with the organization's ability to realize cost savings [3]. Nonetheless, less is known about the impact of the changing healthcare provider landscape on the quality of care in ACOs and their ability to not only realize savings but also earn shared savings back from Medicare. Identifying contributing factors to success in these new value‐based payment arrangements is critical because ACO participants differ in characteristics and structure [3, 4] and most importantly because the Centers for Medicare and Medicaid Services (CMS) plans to have all traditional Medicare beneficiaries in accountable care relationships by 2030 [5].

This study assessed the association between the ACO providers' specialty distribution, quality of care, and the ability of participating organizations to realize savings and earn shared savings during the first decade of the ongoing Medicare Shared Savings Program (MSSP). We also compared these estimated relationships to those in other, more advanced Medicare ACO programs implemented during that time period, but which have ended: the Pioneer and Next Generation ACO (NGACO) models.

## 2 Methods

This study was reviewed and approved by our institutional review board.

### 2.1 Data

The primary data for this study came from the ACO (MSSP) provider‐level research identifiable files (RIFs) and public use files (PUFs) between 2013 and 2021. Because the COVID‐19 pandemic had significant effects on provider access [6], healthcare utilization, and outcomes [7], we analyzed the pre‐ (2013–2019) and post‐COVID‐19 (2020–2021) periods separately. ACO unique identifier numbers were used to link the files. RIFs included information on all national provider identifiers (NPIs) of providers affiliated with the ACO participant, the NPI's specialty codes and their associated taxpayer identification numbers (TINs). For the small proportion of ACO‐TIN‐NPI triad observations (0.5%–3.2% across years) with missing data on NPI, we leveraged the longitudinal nature of the data to impute NPIs for TINs with missing affiliated NPIs. We used the non‐missing data on those TINs' affiliated NPIs in prior and following years, if only one NPI was identified in those years as affiliated with the TINs: < 1% triad observations, overall, remained with missing NPIs after imputation. Similarly, we imputed NPI specialty for NPIs without designated specialty in a given year from data on associated specialty in other surrounding years. The remaining NPIs with missing specialty were categorized as “*unknown*” specialty. Using assignment‐eligible specialties in the MSSP 2021 performance year assignment methodology [8], we categorized provider specialty into four mutually‐exclusive groups: “p*rimary care physicians (PCPs)*,” “n*on‐physician practitioners*,” “*specialists*,” and “o*ther/unknown*.” We also computed the annual percentage of each specialty group within each ACO: “unknown” accounted for 0.9% of ACO‐NPI‐level observations).

We obtained financial and quality data from the ACO performance year financial and quality report PUFs. The PUFs provided data on the ACO's assigned beneficiaries, assigned beneficiary characteristics (gender, race/ethnicity, age category, end‐stage renal disease, disability, dual eligibility for Medicaid), service area, shared savings payment received or losses owed as determined by their earnings/losses, total quality score (Table S1), and financial risk model [8]. For ACOs with a designated primary service area, we identified their primary state of service which we linked to state‐level data on the supply of providers by specialty from the National Physician Compare database (now known as the National Downloadable File), during the study period.

We also used RIFs and PUFs for more advanced ACO programs (which have expired), notably the Pioneer and Next Generation ACO (NGACO) models in secondary analyses.

### 2.2 Variable Descriptions

We considered two outcome measures. The first outcome was the ACO's shared savings/losses per capita, which could be positive or negative ($), as defined by the ratio of the earnings/losses and the total assigned beneficiaries in each performance year. The second outcome was the ACO's performance year overall quality composite score (percentage) based on its performance on metrics spanning four domains including patient/caregiver experience, care coordination/patient safety, preventive health, and at‐risk populations.

ACOs were characterized by the number of providers, the distribution of provider specialty categories, financial risk model, assigned beneficiary characteristics, the supply of providers with Medicare enrollments (total and by specialty) within their primary state of service, shared savings/losses (total and per capita), and quality score.

MSSP participants could elect into either one‐sided financial risk that made them only eligible to earn shared savings payment and not be liable for losses owed, or two‐sided risk, which offered a relatively greater portion of the realized savings as incentive in addition to the liability for owed losses to CMS. For MSSPs, financial risk model election occurs every contract (3 years) period.

### 2.3 Statistical Analyses

We estimated longitudinal ordinary least‐squares (OLS) regression models with random effects, which account for unobserved factors that vary across ACOs and are uncorrelated with the explanatory variables, to assess the association between provider specialty groups and the ACO's outcome of interest.

When modeling changes in per‐capita shared savings earned, regressions were of the general form:

(1) $$\begin{array}{c} Y_{\mathrm{it}}=\beta_{0}+\beta_{1}^{\prime }\text{Prov}\_{\text{group}}_{\mathrm{it}}+\beta_{2}\text{Quality}\_{\text{score}}_{\mathrm{it}}\\ +\beta_{3}\mathrm{two}\_\text{sided}\_{\text{risk}}_{\mathrm{it}}+\delta^{\prime }X_{\mathrm{it}}+\rho_{i}+\tau_{t}+\varepsilon_{\mathrm{it}} \end{array}$$

where $Y_{\mathrm{it}}$ represented shared savings payment receipt/losses owed for ACO i in performance year t. The vector $\text{Prov}\_{\text{group}}_{\mathrm{it}}$ represented indicators for provider specialty categories [e.g., PCP, non‐physician, specialist, other/unknown (the reference)] for MSSPs. ${\hat{\beta }}_{1}$ is the corresponding vector of coefficients that estimated the relationships of interest between provider specialty categories and the outcome variable. The regression also adjusted for the ACO's quality score because shared savings payment can only be earned if the participant met quality standards in addition to having their assigned beneficiaries' expenditures below their specified financial benchmark in that performance year. We also included an indicator for whether the ACO was in a two‐sided risk model. The vector $X_{\mathrm{it}}$ included assigned beneficiary demographics and risk factors, and measures of state‐level supply of healthcare providers by specialty. Beneficiary demographics included sex, age categories, and race‐ethnicity categories, which we controlled for because there are disparities in reports of health status and chronic conditions, healthcare utilization and outcomes across race and ethnicity [9]. Regressions also included ACO's random effect, $\rho_{i}$, assumed to be uncorrelated with the explanatory variables, and year $\left(\tau_{t}\right)$ effects, which adjusted for unobserved factors that changed across years.

A similar multivariable regression model with random effects was estimated using quality score (%) as an outcome in lieu of an explanatory variable. Because ACOs received a pay‐for‐reporting quality score in the first year of participation, indicated by a perfect score (100%) and a pay‐for‐performance quality score afterward, changes in quality score were modeled starting in the ACO's second participation year.

We analyzed the pre‐ and post‐COVID‐19 period separately to evaluate whether the estimated relationships between provider specialty and the outcome variables were different before or after the pandemic. We re‐estimated the main regression analyses for MSSP participants separately by financial risk to evaluate whether the relationships between provider specialties and the outcome variables differed across groups.

In secondary analysis, we compared results from the MSSP to those of Pioneer and NGACOs. Unlike the MSSP, all participants of Pioneer and NGACO models assumed two‐sided financial risk [10]. Recognizing the specificities of each ACO program (e.g., financial benchmark calculation, savings and losses limits) [11], longitudinal OLS regressions with random effects were estimated separately for the MSSP categorized by financial risk model election (2013–2021), Pioneer (2012–2014), and NGACO (2016–2021) programs. The MSSP program is the only one of the three programs that remained active after 2021. Pioneer and NGACO RIFs only provided information on primary care specialty of the provider (not distinguishing physicians from non‐physicians). Unlike the MSSP program, Pioneer and NGACO PUFs also had relatively limited data on assigned beneficiary characteristics (e.g., risk factors).

In sensitivity analyses still focusing on the MSSP, we estimated fixed‐effect regression models that account for unobserved factors assumed to have remained constant over time within ACOs. We also estimated longitudinal logistic regression models to assess the relationship between the likelihood of the ACO participant receiving shared savings earnings and the covariates in the main regression models.

In all regression models, standard errors were clustered at the ACO level to account for correlation between outcome values within an ACO over time. In this study, we conducted 2‐sided statistical tests with *p* < 0.05 considered to be statistically significant and 0.05 ≤ *p* < 0.10 considered to be marginally statistically significant.

## 3 Results

The sample consisted of 865 MSSP ACOs (3860 ACO‐years) (Table 1). Between 2013 and 2021, PCPs represented more than a third (33.90%) of ACOs' workforce, but the percentage of PCPs has decreased across periods. Less than one in five (16.53%) MSSPs were in two‐sided financial risk, with most participants electing greater financial risk since 2019. The average number of assigned Medicare beneficiaries was 18,589. ACO participants had more female (nearly 60%) than male assigned beneficiaries. Beneficiaries aged 0–64 represented 15.56% of MSSP assigned beneficiaries. Beneficiaries with higher risk, as identified by end stage renal disease (ESRD), disability, or being over 65 with dual eligibility for Medicaid and Medicare, constituted less than 25% of assigned beneficiaries, on average, across ACOs. Although the majority of healthcare providers available within states were specialty care providers (> 60%), PCPs and non‐physicians represented the majority of ACOs' provider workforce (57.75%). On average, ACOs earned shared savings payments of $2,358,086, but with relatively higher payments in2020 to 2021 ($4,294,695) than pre‐pandemic, and quality score averaged 93.29% during the study period.

**TABLE 1:** Descriptive characteristics of medicare shared savings program (MSSP) participants (2013–2021).

Variables	2013–2021	2013–2019 (Pre‐COVID)	2020–2021 (Post‐COVID)
	Mean	Mean	Mean
	(SD)	(SD)	(SD)
Number of ACOs	865	747	513
Number of ACO‐years	3860	2872	988
Total number of providers	931	792	1334
	(1484)	(1215)	(2023)
Provider specialty (%)			
Specialists	12.94	13.26	11.98
Primary care physicians (PCPs)	33.90	35.74	28.56
Non‐physician practitioners	23.85	21.26	31.39
Other/unknown	29.31	29.74	28.07
Two‐sided risk model (Yes = 1), %	16.53	8.81	38.97
Number of assigned beneficiaries	18,589	17,951	20,444
	(19,062)	(18,103)	(21,511)
Characteristics of assigned beneficiaries			
Female (%)	57.09	57.19	56.80
Race and ethnicity^f^(%)			
White	83.88	83.57	84.80
Black	9.04	9.37	8.09
Asian	1.94	2.00	1.77
Hispanic	1.87	1.99	1.52
North American native	0.21	0.21	0.20
Other	3.05	2.85	3.62
Age group^f^ (%)			
0–64	15.56	16.62	12.46
65–74	44.75	44.13	46.52
75–84	27.63	27.11	29.15
85+	12.06	12.13	11.88
Risk level^f^ (%)			
ESRD	0.89	0.93	0.76
Disabled	12.70	13.51	10.36
Aged & dual eligibility for medicaid	7.96	8.15	7.40
Aged & nondual	78.45	77.41	81.46
State characteristics			
Total number of providers	41,025	39,406	45,732
	(27,086)	(25,810)	(30,013)
Primary‐care physicians (PCPs)^a^ (%)	17.07	17.74	15.12
Non‐physician practitioners^b^ (%)	19.51	17.92	24.14
Specialty care providers^c^ (%)	63.42	64.34	60.73
Earnings/losses ($)			
Shared savings/losses^d^ ($)	2,358,086	1,691,871	4,294,695
	(4,975,862)	(3,861,723)	(6,955,544)
Per capita shared savings/losses ($)	143	114	228
	(238)	(215)	(279)
Quality Score^e^	93.29	92.90	94.39
	(9.59)	(10.45)	(6.45)

Abbreviations: ACO: accountable care organization; ESRD: end stage renal disease.

^a^ Primary care specialty includes family practice, family medicine, internal medicine, pediatric medicine, general practice, and geriatric medicine.

^b^ Non‐physician practitioners include nurse practitioners, physician assistants, clinical nurse specialists, and certified clinical nurse specialists.

^c^ Specialty care providers may include both physicians and non‐physicians.

^d^ Total earned shared savings payments/owed losses (See Table S1).

^e^ Number of missing (MSSP) = 89 in 2015 for participants in their first performance year in 2015. In performance year 1 of the ACO's first agreement period, the quality score was assigned by the Centers for Medicare and Medicaid Services based on pay for reporting—ACOs received full credit (100%) if all measures were completely reported and less than 100% if one or more measures were not completely reported. Beyond performance year 1 of an ACO's first agreement period, the quality score was determined based on performance against established benchmarks [i.e., (pay for performance) and on quality improvement].

^f^ If an ACO's number of beneficiaries with a specific race/age group/risk factor is missing, we consider the number of this group of beneficiaries to be 0 for the ACO.

**FIGURE 1:**
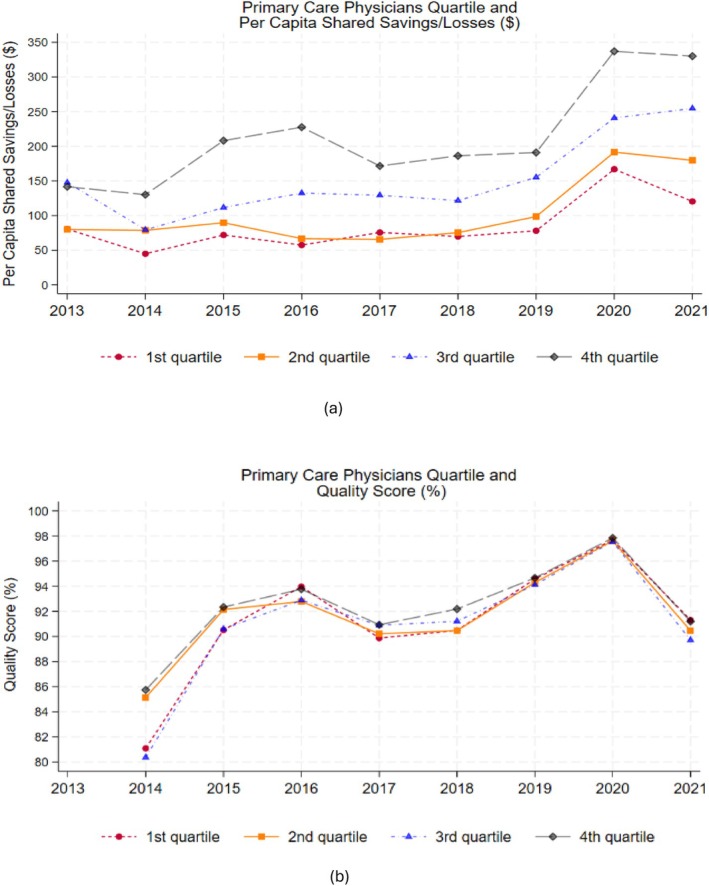
ACO Primary Care Physicians Percentage, Per Capita Shared Savings/Losses and Quality Score in the MSSP (2013–2021). MSSP: Medicare Shared Savings Program. Quartile 1 (bottom/lowest quartile) represents the first quartile with the end of the quartile representing the 25th percentile of the data. Similarly, the end of the second quartile (quartile 2) denotes the 50th percentile, the end of the third quartile (quartile 3) denotes the 75th percentile, and the end of the fourth quartile (quartile 4, top/highest quartile) denotes the 100th percentile. In Performance Year 1 of the ACO's first agreement period, the quality score was assigned by the Centers for Medicare and Medicaid Services based on pay for reporting—ACOs received full credit (100%) if all measures were completely reported and less than 100% if one or more measures were not completely reported. Beyond Performance Year 1 of an ACO's first agreement period, the quality score was determined based on performance against established benchmarks [i.e., (pay for performance) and on quality improvement]. Quality score trends were therefore based on data starting in performance Year 2 for ACOs.

The unadjusted trends in per‐capita shared savings across ACO programs showed that organizations with higher percentages of PCPs, in the 3rd and 4th (top) quartiles, also had relatively higher per‐capita shared savings (Figure 1). However, there was no clear trend in quality scores in relation to the distribution of PCPs across MSSPs. Nonetheless, the increase in quality score across ACOs in 2020 may be due to participants being awarded automatic full credit for some quality measures [12].

Descriptive characteristics of 32 Pioneer and 62 NGACOs showed similarities but also differences across programs compared with MSSPs (Table S2). Pioneer and NGACO participants were relatively larger than MSSPs, as determined by their average assigned beneficiaries: 25,325 in Pioneer and 29,074 in NGACO versus 18,589 in MSSP. ACOs' shared savings earnings were relatively higher in the Pioneer ($2,693,958) and NGACO ($7,324,164 vs. $2,358,086 in the MSSP). Average quality score was comparable across programs: 92.07% in Pioneer and 94.93% in NGACO (vs. 93.29% in MSSP).

### 3.1 Provider Specialty Type and Per‐Capita Shared Savings/Losses

Having higher percentages of PCPs and non‐physicians, in general, was associated with higher per‐capita shared savings payments in ACOs. We found that a 1 percentage‐point increase in the percentages of PCPs and non‐physicians was associated with increases in per‐capita shared savings payment of $2.25 (*p* < 0.01) and $1.82 (*p* = 0.03), respectively, pre‐COVID and $2.73 (*p* < 0.01) and $1.81 (*p* = 0.14) post‐COVID (Table 2, columns 1–2). However, the percentage of specialists was not associated with significant changes in per‐capita shared savings payment. As expected, quality of care was positively associated with shared savings earnings; a 1 percentage‐point increase in quality score was associated with 1.07 to $ 1.08 higher earnings across periods, but the relationship was statistically significant only pre‐pandemic. Compared with ACOs in one‐sided risk, those in two‐sided risk earned $184.10 (*p* < 0.01) more in shared savings payments post‐pandemic.

**TABLE 2:** Association between provider specialty type, ACOs' shared savings received/losses owed and quality of care in the medicare shared savings program (MSSP) (2013–2021).

Variables	(1)	(2)	(3)	(4)
	Per capita shared Savings/losses ($)	Per capita shared Savings/losses ($)	Quality score (%)^a^	Quality score (%)^a^
	2013–2019	2020–2021	2013–2019	2020–2021
% of primary care physicians (PCPs)	2.25***	2.73***	0.10***	−0.02
	(0.47)	(0.95)	(0.03)	(0.02)
% of Non‐physician practitioners	1.82**	1.81	0.09***	−0.05**
	(0.82)	(1.22)	(0.04)	(0.02)
% of specialists	0.25	−1.28	0.11*	−0.10**
	(0.85)	(2.05)	(0.06)	(0.04)
Quality score	1.07***	1.08	—	—
	(0.32)	(1.91)	—	—
Two‐sided risk model (Yes = 1)	26.41	184.10***	3.14***	1.74***
	(38.14)	(21.67)	(1.12)	(0.38)
Assigned beneficiaries' demographics	X	X	X	X
Assigned beneficiaries' risk factors	X	X	X	X
State‐level supply of health care providers by specialty	X	X	X	X
Year aggregate effects	X	X	X	X
Observations (ACO‐years)	2783	988	2125	935
Observations (ACOs)	743	513	708	511

*Note:* One‐sided denotes participation in one‐sided shared savings model: prior to July 2019, this included Track 1, and after July 2019, Level A and Level B. Two‐sided indicates participation in a two‐sided shared savings/losses model for the performance year: prior to July 2019, this included Track 2, Track 3, and Track 1+ and after July 2019, Level C, Level D, Level E, and Enhanced Track. Each column represents a different regression. Longitudinal ordinary‐least‐squares (OLS) regressions were estimated with ACO random effects also adjusted for the ACO's assigned Medicare beneficiaries' demographics including the percentage of female, the percentage of non‐white (the percentage of white was the reference), the percentage of beneficiaries in each age groups (65–74, 75–84, 85+; < 65 as reference), assigned beneficiaries' risk factors including the percentage of beneficiaries over age 65 and with dual eligibility for Medicaid, the percentage of beneficiaries with disability or end‐stage renal disease, the supply of providers (by specialty) in the ACO's primary state of service and year fixed‐effects. Standard errors reported in parentheses were clustered at ACO level. Regression coefficients for covariates are available upon request to the authors. Inference: ***p < 0.01, ** *p* < 0.05, * *p* < 0.1.

Abbreviation: ACO: accountable care organization.

^a^ Regression models assessing the change in quality scores do not include observations in the first participation year of the ACOs because quality scores were determined based on reporting and not performance.

Heterogeneity analysis, separating MSSPs by financial risk model election, suggested that the average results estimated for changes in per‐capita shared savings were driven by relationships among organizations in two‐sided financial risk for the percentage of PCPs but those in one‐sided risk for the percentage of non‐physicians (Table 3, columns 1–2 and 5–6). Unlike in the MSSP, a higher percentage of primary care providers was not associated with statistically significant changes in per‐capita shared savings receipt in Pioneer and NGACO programs (Table S3).

**TABLE 3:** Association between provider specialty type and ACOs' shared savings received/losses owed and quality of care by financial risk (2013–2021).

Variables	(1)	(2)	(5)	(6)	(3)	(4)	(7)	(8)
	MSSP one‐sided				MSSP two‐sided			
	Per capita shared savings/losses ($)	Per capita shared savings/losses ($)	Quality score (%)^a^	Quality score (%)^a^	Per capita shared savings/losses ($)	Per capita shared savings/losses ($)	Quality score (%)^a^	Quality score (%)^a^
	2013–2019	2020–2021	2013–2019	2020–2021	2013–2019	2020–2021	2013–2019	2020–2021
% of Primary care physicians (PCPs)	1.92***	1.70	0.10***	−0.03	5.30***	3.97**	0.04	−0.005
	(0.40)	(1.05)	(0.04)	(0.03)	(1.72)	(1.73)	(0.03)	(0.02)
% of Non‐physician practitioners	2.35***	0.13	0.09**	−0.08**	−1.39	2.99	−0.01	−0.05
	(0.76)	(1.33)	(0.04)	(0.03)	(2.43)	(2.22)	(0.04)	(0.03)
% of Specialists	0.50	−1.89	0.11	−0.11*	−5.68	−1.08	0.07	−0.12**
	(0.85)	(2.06)	(0.06)	(0.05)	(4.30)	(4.22)	(0.09)	(0.06)
Quality score	1.07***	2.95*	—	—	2.92	−4.14	—	—
	(0.31)	(1.63)	—	—	(3.60)	(5.63)	—	—
Assigned beneficiaries' demographics	X	X	X	X	X	X	X	X
Assigned beneficiaries' risk factors	X	X	X	X	X	X	X	X
State‐level supply of health care providers by specialty	X	X	X	X	X	X	X	X
Year aggregate effects	X	X	X	X	X	X	X	X
Observations (ACO‐years)	2530	603	1918	565	253	385	207	370
Observations (ACOs)	697	323	669	319	113	208	104	207

*Note:* One‐sided denotes participation in one‐sided shared savings model: prior to July 2019, this included Track 1, and after July 2019, Level A and Level B. Two‐sided indicates participation in a two‐sided shared savings/losses model for the performance year: prior to July 2019, this included Track 2, Track 3, and Track 1+ and after July 2019, Level C, Level D, Level E, and Enhanced Track. Each column represents a different regression for each of the groups labeled MSSP one‐sided and MSSP two‐sided across periods (2013–2019 and 2020–2021). Longitudinal ordinary‐least‐squares (OLS) regressions were estimated with ACO random effects also adjusted for the ACO's assigned Medicare beneficiaries' demographics including the percentage of female, the percentage of non‐white (the percentage of white was the reference), the percentage of beneficiaries in each age groups (65–74, 75–84, 85+; < 65 as reference), assigned beneficiaries' risk factors including the percentage of beneficiaries over age 65 and with dual eligibility for Medicaid, the percentage of beneficiaries with disability or end‐stage renal disease, the supply of providers (by specialty) in the ACO's primary state of service and year fixed‐effects. Standard errors reported in parentheses were clustered at ACO level. Regression coefficients for covariates are available upon request to the authors. Inference: ***p < 0.01, ** *p* < 0.05, * *p* < 0.1.

Abbreviations: ACO: accountable care organization; MSSP: Medicare Shared Savings Program.

^a^ Regression models assessing the change in quality scores do not include observations in the first participation year of the ACOs because quality scores were determined based on reporting and not performance in performance year 1.

### 3.2 Provider Specialty Type and Quality Score

Similar to findings with per‐capita shared savings payments, higher percentages of PCPs and non‐physicians were associated with higher quality scores in MSSP only pre‐pandemic. Notably, a 1 percentage‐point increase in the percentages of PCPs and non‐physicians was associated with higher quality scores of 0.10 percentage points (*p* < 0.01) and 0.09 percentage points (*p* < 0.01), respectively (Table 2, columns 3–4). A 1 percentage‐point increase in the percentage of specialists was also associated with a 0.11 percentage point (*p* = 0.08) increase in quality score pre‐pandemic. These relationships were different post‐pandemic: A 1 percentage‐point increase in the percentages of non‐physicians and specialists was associated with lower quality scores of 0.05 percentage points (*p* = 0.02) and 0.10 percentage points (*p* = 0.02), respectively. Two‐sided risk was also positively and significantly associated with quality score across periods.

Analyses across financial risk models suggested that the estimated relationships between provider specialty distribution and quality score were driven by MSSPs in one‐sided financial risk. (Table 3, columns 3–4 and 7–8). Unlike in the MSSP, higher percentages of primary care providers were not associated with better quality scores in Pioneer and NGACO (Table S3).

### 3.3 Sensitivity Analyses

Focusing on MSSPs, the associations estimated in the random‐effect models between provider specialty type and the outcome variables remained similar in direction and magnitude, in general, though with lower statistical significance in the ACO‐fixed‐effect models (Table S4).

We also estimated that a 1‐percentage point increase in the percentage of PCPs was associated with a higher likelihood of earning shared savings of 0.34 percentage points (*p* < 0.01) pre‐pandemic and of 0.53 percentage points (*p* < 0.01) post‐pandemic, respectively, on average, among MSSP participants (Table S5). A 1‐percentage point increase in the percentage of non‐physicians was associated with an increase in the likelihood of earning shared savings of 0.21 percentage points (*p* = 0.09) only pre‐pandemic.

## 4 Discussion

To our knowledge, this is the first study to investigate the association between provider workforce composition and performance as measured by shared savings earnings and quality scores in Medicare ACOs during the first decade of the ACO program. We assessed these relationships separately by ACO program to avoid masking potential differences across programs.

We found in this study that PCPs represented over a third of providers in MSSPs, while representing about 17% of providers in general across states. This is consistent with this value‐based program emphasizing primary care and care coordination. A high percentage of primary care providers in value‐based care is critical because primary care serves as a first point of contact when seeking care and is “the foundation of a high‐functioning health care system.” [13]

Multivariable longitudinal regression analyses suggested that higher percentages of PCPs and non‐physicians were associated with higher per‐capita shared savings and quality scores in ACOs participating in the largest and ongoing MSSP. These results were further corroborated by the associations estimated between changes in PCPs and the likelihood of earning shared savings payments. The null results found when estimating the relationships between the percentage of primary care providers and the outcome variables in the expired Pioneer and NGACO programs could be explained in part by the analyses not adjusting for the participants' assigned beneficiary risk factors—whose data were unavailable for these programs. The differences in findings observed across programs may also be owing to programmatic differences. The Pioneer and NGACO models assumed shared savings/losses arrangements with relatively higher levels of risks and rewards than the MSSP [14, 15]. Beneficiary alignment was done prospectively in Pioneer and NGACOs versus retrospectively in MSSPs, in general [16, 17]. Researchers found higher average per‐capita Medicare spending, which likely reduces the likelihood of earning shared savings if spending changes unfavorably relative to the benchmark, among ACOs with patients assigned prospectively relative to retrospectively assigned patients [18]. Furthermore, the Pioneer program, a single “*closed*” cohort [19] unlike the MSSP and NGACO, was evaluated for a shorter period in this study, 3 years, and early into the implementation of the ACO programs, where participants still experimented with different approaches to care management for their sicker patients [20]. Although expired, evaluation of these programs was important because some participants in these more advanced programs have transitioned into other ACO programs (e.g., MSSP).

Although our findings suggest that a higher proportion of PCPs is associated with an increase in per‐capita shared savings, shared savings payments are different from actual healthcare cost savings. Prior literature suggested that while shared savings payments were below estimated gross healthcare spending reductions between 2013 and 2019, they exceeded gross spending reductions in 2020–2021 [21]. Furthermore, shared savings payment determinations are based on the ACOs' pre‐determined financial benchmarks, which may be different from what would have occurred in the absence of the ACO program (i.e., a counterfactual). These financial benchmarks are calculated based on a blend of regional and historical spending of the ACO, and inflation and patient risk adjustments, but do not entirely account for potential shocks that affect healthcare spending beyond the ACO's region (e.g., nationwide) [22]. If financial benchmarks do not also account for changes in the national spending trend, ACOs could be rewarded with shared savings payments in periods with unexpected spending reductions outside of its control (e.g., COVID‐19 pandemic) [22]. Notably, the COVID‐19 pandemic shocked the nation's entire healthcare system [23], creating gaps in provider access [24], healthcare utilization, and therefore spending nationwide, but we observed an increase across ACOs in per‐capita shared savings earnings in 2020 (Figure 1). Furthermore, additional considerations need to be taken about the voluntary participation (and exit) of organizations into (or from) the ACO programs [25, 26, 27], organizational structure [4, 28], strategic changes to affiliated practices, and Medicare beneficiary attribution patterns [29], when assessing the impact of ACOs on healthcare spending in general.

Notwithstanding the difference in estimated relationships across ACO programs, findings in the largest and permanent MSSP program are in line with earlier findings indicating the importance of advanced primary care in the success of ACOs with patient‐centered medical homes status [30, 31]. Our results also reiterate the importance of strengthening primary care to address the vulnerabilities of our healthcare system [32] and ultimately bending the cost curve in the US [33, 34].

The growing reliance of healthcare systems on non‐medical doctors may be for lower reimbursements [35] and may help address the growing demand for and current shortage of physicians in the US [36]. However, the growing reliance on non‐medical doctors should continue to support primary care to improve access to health care, particularly for underserved patients, continuity of care for chronically ill patients, and for less complex healthcare needs of patients [37, 38, 39]. ACO participants with a higher percentage of non‐primary care providers may want to increase the primary care orientation of their workforce specialty or invest in efforts to reduce potential leakages [40] that hinder the ACO's ability to manage their assigned beneficiary spending [41, 42].

Our findings also have implications for the newly implemented ACO Primary Care Flex Model (ACO PC Flex Model), which will increase funding for primary care in ACOs [13]. Future evaluation of these programs would be necessary to show whether and how health outcomes, quality, and costs of care are impacted by these new incentives.

The statistically insignificant relationships estimated among MSSPs in two‐sided financial risk pre‐pandemic may be due to the relatively smaller number of participants in that group. However, the recent program overhaul, “Pathways to Success” [43], offering more options to transition into two‐sided risk more quickly explains the large increase in the proportion of ACOs in two‐sided risk in 2020–2021. Continuous monitoring of the program is needed to show if more financial accountability will have the desired effect on performance of these organizations.

Findings from this study should be considered in light of some limitations. First, only data on Medicare ACOs were analyzed, and results may not be generalizable to non‐ACOs or organizations with ACO contracts from non‐Medicare payers and other value‐based payment models with less emphasis on primary care. We have assessed changes in overall quality score—used to determine whether ACOs are eligible to earn shared savings—in relation to the provider workforce composition of ACOs. Though the estimated relationships may mask heterogeneities across different quality metrics, analyzing changes in nearly 30 individual quality measures is beyond the scope of this study. We only analyzed 2 years of data post‐pandemic, but our analyses suggesting differences in the estimated relationships between provider specialty, shared savings payment earnings, and quality score provide additional evidence for the differences across pre‐ and post‐pandemic periods. As utilization [44] and healthcare employment rebounded from the pandemic by 2024 [45], future studies should continue to assess these relationships.

## 5 Conclusion

Higher percentages of PCPs and non‐physicians were associated with higher per‐capita earned shared savings and quality scores among MSSPs. As value‐based payment models continue to expand, continuous monitoring should occur, particularly for those models that provide greater incentives for primary care to determine their ability to further improve care efficiency and find out whether more or better incentives are needed.

## Disclosure

The authors have nothing to report.

## Conflicts of Interest

The authors declare no conflicts of interest.

## Supporting information


**Data S1:** Supplementary Information.

## Data Availability

The data that support the findings of this study are available from Centers for Medicare and Medicaid Services. Restrictions apply to the availability of these data, which were used under license for this study. Data are available from the author(s) with the permission of Centers for Medicare and Medicaid Services.
