# Supplementary material for: Clinician Specialties, Quality Score and Shared Savings Receipt in Accountable Care Organizations
Source: Health Serv Res. 2025 Sep 4;61(1):e70033. doi: 10.1111/1475-6773.70033 (PMC12857478; doi:10.1111/1475-6773.70033)
Supplement: Supplementary file 1 — Data S1: Supplementary Information. [file HESR-61-e70033-s001.docx]

**Clinician specialties, quality score and shared savings receipt in Accountable Care Organizations**

**Ouayogodé and Liang, 2025**

**Appendix**

**Table.A1:** Definitions of Outcome Measures Based on the Accountable Care Organization Performance Year Financial and Quality Results Public Use File Data Dictionary

| **Outcome measure** | **Term name in ACO data file** | **Description** |
| --- | --- | --- |
| Shared Savings/Losses | Earned Shared Savings Payments/Owed Losses | *Total earned shared savings:* The ACO’s share of savings for ACOs whose savings rate equaled or exceeded their minimum savings rate (MSR), and who were eligible for a performance payment because they met the program’s quality performance standard. This amount accounts for the application of the ACO’s final sharing rate based on quality performance (based on ACO track), as well as the reduction in performance payment due to sequestration and application of the performance payment limit. This amount does not account for repayment of advance payments.  *Total earned shared losses:* The ACO’s share of losses for ACOs in two-sided tracks whose losses rate equaled or exceeded their minimum loss rate (MLR), which is the negative of the MSR chosen. This amount accounts for the application of the ACO’s final loss sharing rate based on quality performance (based on ACO track), the loss sharing limit and the Extreme and Uncontrollable Circumstance adjustment. |
| Quality Score | Quality Score | In Performance Year 1 of an ACO’s first agreement period, the quality score is 100% if all measures were completely reported and less than 100% if one or more measures were not completely reported. Beyond Performance Year 1 of an ACO’s first agreement period, the quality score will be determined not only by whether all measures were completely reported but also on their performance against established benchmarks and on quality improvement.  For ACOs determined to have been affected by an Extreme and Uncontrollable Circumstance, the quality score is the higher of the ACO's calculated initial quality score or the national mean quality score across all Shared Savings Program ACOs who met the quality performance standard before application of the 'extreme and uncontrollable circumstances' policy. |

Abbreviation: ACO=Accountable Care Organization

**Source**: Adapted by the Authors from the 2021 Centers for Medicare and Medicaid Services Medicare Shared Savings Program Performance Year Financial and Quality Results Public Use File data dictionary.

**Table.A2:** Descriptive Characteristics of Medicare ACO Participants (2012-2021)

| **Variables** | **MSSP (2013-2021)** | **Pioneer (2012-2014)** | **NGACO (2016-2021)** |
| --- | --- | --- | --- |
|  | Mean (sd) | Mean (sd) | Mean (sd) |
| **Number of ACOs** | 865 | 32 | 62 |
| **Number of ACO-years** | 3,860 | 75 | 225 |
| **Total Number of Providers** | 931 | 826 | 1,124 |
|  | (1484) | (663) | (1133) |
| **Provider Specialty, %** |  |  |  |
| Specialists | 12.94 | - | - |
| Primary Care Physicians (PCPs) | 33.90 | - | - |
| Non-Physician Practitioners | 23.85 | - | - |
| Other/Unknown | 29.31 | - | - |
|  |  |  |  |
| Primary Care Providers | 57.75 | 59.84 | 53.26 |
|  |  |  |  |
| **Two-sided Risk Model (Yes=1), %** | 16.53 | - | - |
| **Number of Assigned Beneficiaries** | 18,589 | 25,325 | 29,074 |
|  | (19,062) | (16,684) | (21,643) |
| **Characteristics of Assigned Beneficiaries** |  |  |  |
| **Female, %** | 57.09 | 58.93 | 57.36 |
| **Race and Ethnicity*, %** |  |  |  |
| White | 83.88 | - | - |
| Black | 9.04 | - | - |
| Asian | 1.94 | - | - |
| Hispanic | 1.87 | - | - |
| North American Native | 0.21 | - | - |
| Other | 3.05 | - | - |
| **Age Group*, %** |  |  |  |
| 0-64 | 15.56 | 14.87 | 11.50 |
| 65-74 | 44.75 | - | - |
| 75-84 | 27.63 | - | - |
| 85+ | 12.06 | - | - |
|  |  |  |  |
| 65+ | 84.44 | 85.13 | 88.50 |
| **Risk Level*, %** |  |  |  |
| ESRD | 0.89 | 0.91 | - |
| Disabled | 12.70 | 22.14 | - |
| Aged & Dual Eiligbility for Medicaid | 7.96 | - | - |
| Aged & NonDual | 78.45 | - | - |
| **State Characteristics** |  |  |  |
| Total Number of Providers | 41,025 |  |  |
|  | (27,086) |  |  |
| Primary-care Physicians (PCPs)^A^, % | 17.07 |  |  |
| Non-Physician Practitioners^B^, % | 19.51 |  |  |
| Specialty Care Providers^C^,% | 63.42 |  |  |
| **Earnings/Losses, $** |  |  |  |
| Shared Savings/Losses ^D^, $ | 2,358,086 | 2,693,958 | 7,324,164 |
|  | (4,975,862) | (4,801,525) | (10,465,868) |
| Per Capita Shared Savings /Losses, $ | 143 | 112 | 286 |
|  | (238) | (188) | (390) |
| Quality Score ^E^ | 93.29 | 92.07 | 94.93 |
|  | (9.59) | (8.09) | (7.76) |

Abbreviations: ACO-Accountable Care Organization; ESRD-End state renal disease; MSSP-Medicare Shared Savings Program; NGACO-Next Generation ACO

***Notes***: Pioneer ACO and NGACO primary care providers were those with primary care provider indicators in research identifiable files. Pioneer ACO's primary care provider indicator information was missing in 2012 research identifiable files. It was imputed based on 2013-2014 Pioneer research identifiable files and MSSP ACO research identifiable files. The percentage of primary care providers was calculated as the ratio of the number of primary care providers to all providers who were reported to have participated in an ACO (excluding preferred providers for NGACO).

^A^ Primary care specialties includes family practice, family medicine, internal medicine, pediatric medicine, general practice, and geriatric medicine.

^B^ Non-physician practitioners include nurse practitioners, physician assistants, clinical nurse specialists, certified clinical nurse specialists.

^C^ Specialty care providers may include both physicians and non-physicians.

^D^ Total earned shared savings payments/owed losses (*See Appendix Table1*).

^E^ Number of missing (MSSP)=89 in 2015 for participants in their first performance year in 2015. In performance year 1 of the ACO’s first agreement period, the quality score was assigned by the Centers for Medicare and Medicaid Services based on pay for reporting—ACOs received full credit (100%) if all measures were completely reported and less than 100% if one or more measures were not completely reported. Beyond performance year 1 of an ACO’s first agreement period, the quality score was determined based on performance against established benchmarks (i.e. (pay for performance) and on quality improvement.

**Table.A3**: Association between Provider Specialty Type and ACOs' Shared Savings Received /Losses Owed and Quality of Care by ACO Program (2012-2021)

|  | **(1)** | **(2)** | **(3)** | **(4)** | **(5)** | **(6)** | **(7)** | **(8)** | **(9)** | **(10)** |
| --- | --- | --- | --- | --- | --- | --- | --- | --- | --- | --- |
|  | **MSSP (2013-2021)** | | **MSSP (one-sided) (2013-2021)** | | **MSSP (two-sided) (2013-2021)** | | **Pioneer (2012-2014)** | | **NGACO (2016-2021)** | |
| VARIABLES | **Per Capita Shared Savings/Losses ($)** | **Quality Score (%)†** | **Per Capita Shared Savings/Losses ($)** | **Quality Score (%)†** | **Per Capita Shared Savings/Losses ($)** | **Quality Score (%)†** | **Per Capita Shared Savings/Losses ($)** | **Quality Score (%)†** | **Per Capita Shared Savings/Losses ($)** | **Quality Score (%)†** |
|  |  |  |  |  |  |  |  |  |  |  |
| % of Primary Care Physicians (PCPs) | 2.28*** | 0.09*** | 1.97*** | 0.07** | 5.34*** | 0.01 | _ | _ | _ | _ |
|  | (0.43) | (0.03) | (0.39) | -0.03 | -1.59 | (0.02) | _ | _ | _ | _ |
| % of Non-Physician Practitioners | 1.64** | 0.07** | 1.35** | 0.10** | 2.75 | -0.02 | _ | _ | _ | _ |
|  | (0.68) | (0.03) | (0.66) | (0.04) | (1.75) | (0.02) | _ | _ | _ | _ |
| % of Specialists | -0.20 | 0.09* | -0.34 | 0.10 | -1.40 | -0.09 | _ | _ | _ | _ |
|  | (0.83) | (0.05) | -0.82 | -0.06 | (3.29) | -0.05 | _ | _ | _ | _ |
| % of Primary Care Providers | _ | _ | _ | _ | _ | _ | -0.72 | -0.02 | 3.06 | -0.05 |
|  | _ | _ | _ | _ | _ | _ | (0.74) | -0.03 | (1.86) | (0.04) |
| Quality Score | 1.15*** | _ | 0.91*** | _ | -2.00 | _ | -7.23** | _ | 5.34** | _ |
|  | (0.34) | _ | (0.32) | _ | (3.93) | _ | (3.66) | _ | (2.25) | _ |
| Two-sided Risk Model (Yes=1) | 134.48*** | 1.10*** | _ | _ | _ | _ | _ | _ | _ | _ |
|  | (19.03) | (0.33) | _ | _ | _ | _ | _ | _ | _ | _ |
|  |  |  |  |  |  |  |  |  |  |  |
| Assigned Beneficiaries' Demographics | X | X | X | X | X | X | X | X | X | X |
| Assigned Beneficiaries' Risk Factors | X | X |  |  |  |  |  |  |  |  |
| State-level Supply of Health Care Providers by Specialty | X | X |  |  |  |  |  |  |  |  |
| Year Aggregate Effects | X | X | X | X | X | X | X | X | X | X |
| Observations (ACO-years) | 3,771 | 3060 | 3,133 | 2,483 | 638 | 577 | 75 | 43 | 225 | 163 |
| Observations (ACOs) | 861 | 824 | 777 | 745 | 242 | 232 | 32 | 23 | 62 | 51 |

Abbreviations: ACO-Accountable Care Organization; MSSP -Medicare Shared Savings Program; NGACO-Next Generation Accountable Care Organization.

***Notes***: One-sided denotes participation in one-sided shared savings model: prior to July 2019, this included Track 1, and after July 2019, Level A and Level B. Two-sided indicates participation in a two-sided shared savings/losses model for the performance year: prior to July 2019, this included Track 2, Track 3, and Track 1+ and after July 2019, Level C, Level D, Level E, and Enhanced Track.

Each column represents a different regression for each of the groups labeled MSSP one-sided (2013-2021), MSSP two-sided (2013-2021), Pioneer ACO (2012-2014) and NGACO (2016-2021). Longitudinal ordinary-least-squares (OLS) regressions were estimated with random effects and adjusted for the ACO’s assigned Medicare beneficiaries’ demographics, and year fixed-effects. Due to limited data on beneficiary characteristics in the Pioneer and NGACO datasets, assigned beneficiaries’ demographics included in regressions were the percentages of female and beneficiaries over 65.

Pioneer ACO and NGACO primary care providers were those with primary care provider indicators in research identifiable files. Pioneer ACO's primary care provider indicator information was missing in 2012 research identifiable files. It was imputed based on 2013-2014 Pioneer research identifiable files and MSSP ACO research identifiable files. The percentage of primary care providers was calculated as the ratio of the number of primary care providers to all providers who were reported to have participated in an ACO (excluding preferred providers for NGACO).

†Regression models assessing the change in quality scores do not include observations in the first participation year of the ACOs because quality scores were determined based on reporting and not performance in performance year 1.

Standard errors reported in parentheses were clustered at ACO level. Regression coefficients for covariates are available upon request to the authors. Inference: *** p<0.01, ** p<0.05, * p<0.1.

**Table.A4:** Association between Provider Specialty Type and ACOs' Performance in the Medicare Shared Savings Program (MSSP) (2013-2021)

|  | **(1)** | **(2)** | **(3)** | **(4)** | **(5)** | **(6)** | **(7)** | **(8)** |
| --- | --- | --- | --- | --- | --- | --- | --- | --- |
|  | **(2013-2019)** | | **(2020-2021)** | | **(2013-2019)** | | **(2020-2021)** | |
|  | **RE** | **FE** | **RE** | **FE** | **RE** | **FE** | **RE** | **FE** |
| VARIABLES | **Per Capita Shared Savings/Losses ($)** | | **Per Capita Shared Savings/Losses ($)** | | **Quality Score (%)†** | | **Quality Score (%)†** | |
|  |  |  |  |  |  |  |  |  |
| % of Primary Care Physicians (PCPs) | 2.25*** | 2.00** | 2.73*** | 3.64 | 0.10*** | 0.10** | -0.02 | -0.11 |
|  | (0.47) | (0.77) | (0.95) | (3.25) | (0.03) | (0.04) | (0.02) | (0.11) |
| % of Non-Physician Practitioners | 1.82** | 0.83 | 1.81 | -2.06 | 0.09*** | 0.11** | -0.05** | 0.11 |
|  | (0.82) | (1.64) | (1.22) | (4.90) | (0.04) | (0.05) | (0.02) | (0.17) |
| % of Specialists | 0.25 | 0.21 | -1.28 | 2.79 | 0.11* | -0.00 | -0.10** | -0.28 |
|  | (0.85) | (1.26) | (2.05) | (7.06) | (0.06) | (0.06) | (0.04) | (0.27) |
| Quality Score | 1.07*** | 1.27** | 1.08 | 0.30 | - | - | - | - |
|  | (0.32) | (0.52) | (1.91) | (2.01) | - | - | - | - |
| Two-sided Risk Model (Yes=1) | 26.41 | -0.09 | 184.10*** | -18.98 | 3.14*** | 2.63** | 1.74*** | 4.97*** |
|  | (38.14) | (53.48) | (21.67) | (55.42) | (1.12) | (1.12) | (0.38) | (1.18) |
| Assigned Beneficiaries' Demographics | X | X | X | X | X | X | X | X |
| Assigned Beneficiaries' Risk Factors | X | X | X | X | X | X | X | X |
| State-level Care Provider Composition (National Physician Compare) | X | X | X | X | X | X | X | X |
| Year Aggregate Effects | X | X | X | X | X | X | X | X |
| Observations (ACO-years) | 2,783 | 2,783 | 988 | 988 | 2,125 | 2,125 | 935 | 935 |
| Observations (ACOs) | 743 | 743 | 513 | 513 | 708 | 708 | 511 | 511 |

Abbreviations: ACO- Accountable Care Organization; RE-regression with ACO random effect; FE-regression with ACO fixed effect.

***Notes***: One-sided denotes participation in one-sided shared savings model: prior to July 2019, this included Track 1, and after July 2019, Level A and Level B. Two-sided indicates participation in a two-sided shared savings/losses model for the performance year: prior to July 2019, this included Track 2, Track 3, and Track 1+ and after July 2019, Level C, Level D, Level E, and Enhanced Track.

Regressions adjusted for the ACO’s assigned Medicare beneficiaries’ demographics including the percentage of female, the percentage of non-white (the percentage of white was the reference), the percentage of beneficiaries in each age groups (65-74, 75-84, 85+; <65 as reference), assigned beneficiaries’ risk factors including the percentage of beneficiaries over age 65 and with dual eligibility for Medicaid, the percentage of beneficiaries with disability or end-stage renal disease, the supply of providers (by specialty) in the ACO's primary state of service and year fixed-effects.

^†^ Regression models assessing the change in quality scores do not include observations in the first participation year of the ACOs because quality scores were determined based on reporting and not performance.

Standard errors were clustered at ACO level and are in parentheses. Regression coefficients for covariates are available upon request to the authors. Inference: *** p<0.01, ** p<0.05, * p<0.1

**Table.A5:** Association between Provider Specialty Type and the Likelihood of ACOs Receiving Shared Savings in the Medicare Shared Savings Program (MSSP) (2013-2021)

|  | **(2013-2019)** | **(2020-2021)** |
| --- | --- | --- |
| VARIABLES | **(1)** | **(2)** |
|  |  |  |
| % of Primary Care Physicians (PCPs) | 0.0034*** | 0.0053*** |
|  | (0.0009) | (0.0017) |
| % of Non-Physician Practitioners | 0.0021* | -0.0006 |
|  | (0.0013) | (0.0020) |
| % of Specialists | 0.0002 | -0.0043 |
|  | (0.0019) | (0.0038) |
| Quality Score | 0.0022*** | 0.0055** |
|  | (0.0008) | -0.0027 |
| Two-sided Risk Model | 0.1738*** | 0.2545*** |
|  | -0.0619 | -0.0342 |
| Assigned Beneficiaries' Demographics | X | X |
| Assigned Beneficiaries' Risk Factors | X | X |
| State-level Supply of Health Care Providers by Specialty | X | X |
| Year Aggregate Effects | X | X |
| State Effects | X | X |
| State Linear Time Trends | X | X |
| Observations (ACO-years) | 2,783 | 988 |
| Observations (ACOs) | 743 | 513 |

Abbreviations: ACO-Accountable Care Organization

***Notes***: One-sided denotes participation in one-sided shared savings model: prior to July 2019, this included Track 1, and after July 2019, Level A and Level B. Two-sided indicates participation in a two-sided shared savings/losses model for the performance year: prior to July 2019, this included Track 2, Track 3, and Track 1+ and after July 2019, Level C, Level D, Level E, and Enhanced Track.

Each column represents a different regression. Longitudinal logistic regressions estimated with ACO random effects, adjusted for the ACO’s assigned Medicare beneficiaries’ demographics including the percentage of female, the percentage of non-white (the percentage of white was the reference), the percentage of beneficiaries in each age groups (65-74, 75-84, 85+; <65 as reference), assigned beneficiaries’ risk factors including the percentage of beneficiaries over age 65 and with dual eligibility for Medicaid, the percentage of beneficiaries with disability or end-stage renal disease, year fixed-effects, the ACO’s primary state of service (the state with the most assigned beneficiaries) fixed-effects and primary state linear time trends. Marginal effects from the regressions are reported.

Standard errors reported in parentheses were clustered at ACO level. Regression coefficients for covariates are available upon request to the authors. Significantly different from 0 in a two-sided test at the *10%, **5%, ***1% level.
